# The Nemesis of Folly

**Published:** 1923-08

**Authors:** 


### THE NEMESIS OF FOLLY.

CURSES and chickens are by no means the only things that come home to roost. Sooner or later all human folly does the same, and it very rarely happens that the evil consequences can be confined to the author of the folly. We are the creatures of each other, and the best and wisest and most innocent have to bear a share of the results of other people's stupidity or wrongdoing. The serious outbreak of small-pox at Gloucester, and the smaller epidemics elsewhere, are very apt cases in point. Gloucester, like many other towns, is, broadly speaking, unvaccinated, and it is now suffering from consequences which were virtually certain to arrive, sooner or later. The exigencies of monthly publication preclude us from quoting figures which vary from day to day, and it must be enough to say that the number of cases at Gloucester which have been diagnosed as small-pox has been so large that the utmost anxiety is reasonably felt as to when, and where, the epidemic will end. These diagnoses are not those of inexperienced young practitioners who have had little, if any, opportunity of observing well-marked cases of this terrible disease, but of highly trained specialists who know very well the difference between chicken-pox and small-pox. The suggestion that Gloucester and Cheltenham, and other places, are having to contend with nothing worse than outbreaks of chicken-pox is too absurd to be discussed. We prefer the evidence of the experts to the theories of the prejudiced.

We have no intention of entering upon the futile and exhausted controversy about the efficacy of inoculation as a preventive of small-pox. That is a closed issue in the minds not only of most medical men but of most persons capable of weighing the value of evidence. The fact that a sprinkling of doctors and of perfectly intelligent and instructed lay people are either doubtful of the virtue of vaccination or are satisfied that it has none need not detain us—no cause was ever so forlorn as entirely to lack adherents. Taking it, then, as established that the average vaccinated person is armoured against small-pox and that the average unvaccinated person is an invitation to it, the grave fact with which we have to deal is that only about one-third of the people of this country possess a defence which experience has shown to be, as a rule, adequate. The other two-thirds are walking about unvaccinated, either because their parents were "conscientious objectors," or were merely indifferent, or because they have themselves given the matter very little thought. We have no desire to be harsh towards "conscientious objectors," but we are satisfied that very often their objections are rooted in politics, using the word in the largest sense, rather than in conscience. They represent an attitude of mind rather than profound conviction. But, however that may be, they are a proved danger to the community, and the time has come when vaccination ought once more to be made compulsory. It is absurd to make the notification of certain diseases compulsory, while allowing the careless, the callous, or the fanatical to prepare the ground for one of the worst of those diseases.

If and when we do this, however, it would only be reasonable that we should, so far as we can, cut the ground from under the feet of objectors. Complaints are made, for instance, of the quality and freshness of the lymph available to the ordinary medical practitioner. Why should Government lymph be reserved solely for Public Vaccinators? Why, again, should not doctors be more vocal in their denunciation of the dangers of neglecting vaccination? The truth is that we have all become rather slack in the matter. Now we are sharply pulled up not by an epidemic of merely "mild" cases, but of cases severe enough to kill. We have grown accustomed to deaths from small-pox, but they will speedily become only too familiar if it continues to be as easy to obtain exemption from vaccination as it is to secure the dole. Above all, let it be clearly understood that, properly speaking, there is no such thing as a "mild" epidemic of this loathsome disease—we might as well speak of a mild and gentle tiger. At any moment the one, like the other, may become savage and deadly.

